# Retraction: Yizhi Qingxin formula extract ameliorates cognitive decline in aged rats via the brain-derived neurotrophic factor/tropomyosin receptor kinase B pathway

**DOI:** 10.3389/fphar.2024.1451575

**Published:** 2024-07-09

**Authors:** Frontiers Editorial Office

The journal retracts the 30 April 2020 article cited above. Following publication, concerns were raised regarding the integrity of the images in the published figures. The authors failed to provide a satisfactory explanation during the investigation, which was conducted in accordance with Frontiers’ policies. As a result, the data and conclusions of the article have been deemed unreliable and the article has been retracted.

This retraction was approved by the Chief Executive Editor of Frontiers. The authors received a communication regarding the retraction and had a chance to respond. This communication has been recorded by the publisher.

